# Simulation and Experiment for Growth of High-Quality and Large-Size AlN Seed Crystals by Spontaneous Nucleation

**DOI:** 10.3390/s20143939

**Published:** 2020-07-15

**Authors:** Zuoyan Qin, Wenhao Chen, Danxia Deng, Zhenhua Sun, Baikui Li, Ruisheng Zheng, Honglei Wu

**Affiliations:** Key Laboratory of Optoelectronic Devices and Systems of Ministry of Education and Guangdong Province, College of Physics and Optoelectronic Engineering, Shenzhen University, Shenzhen 518060, China; 2176285305@email.szu.edu.cn (Z.Q.); chenwenhao2017@email.szu.edu.cn (W.C.); 1800282005@email.szu.edu.cn (D.D.); szh@szu.edu.cn (Z.S.); libk@szu.edu.cn (B.L.); rszheng@szu.edu.cn (R.Z.)

**Keywords:** bulk AlN crystal, simulation, spontaneous nucleation, single growth mode, nucleation control, temperature gradient

## Abstract

Seed crystals are the prerequisite for the growth of high quality and large size aluminum nitride (AlN) single crystal boules. The physical vapor transport (PVT) method is adopted to grow AlN seed crystal. However, this method is not available in nature. Herein, the temperature field distribution in the PVT furnace was simulated using the numerical analysis method to obtain free-standing and large-size seeds. The theoretical studies indicate that the temperature distribution in the crucible is related to the crucible height. According to the theory of growth dynamics and growth surface dynamics, the optimal thermal distribution was achieved through the design of a specific crucible structure, which is determined by the ratio of top-heater power to main-heater power. Moreover, in our experiment, a sole AlN single crystal seed with a length of 12 mm was obtained on the tungsten (W) substrate. The low axial temperature gradient between material source and substrate can decrease the nucleation rate and growth rate, and the high radial temperature gradient of the substrate can promote the expansion of crystal size. Additionally, the crystallinity of the crystals grown under different thermal field conditions are analyzed and compared. The Raman results manifest the superiority of the thermal inversion method in the growth of high quality AlN single crystal.

## 1. Introduction

The single-crystalline aluminum nitride (AlN) is a highly promising semiconductor material with an ultra-wide direct bandgap of 6.2 eV [1]. Therefore, AlN plays an important role in deep-ultraviolet devices, such as the light emission device [2], which has a great potential in sensor [3,4], water purification [5], non-line-of-sight communication [6], etc. Specifically, the quality improvement of the AlN single crystal is beneficial to the development and performance enhancement of deep-ultraviolet and even vacuum-ultraviolet photodetectors [7,8]. Owing to the advantage of excellent electrical properties, high temperature and pressure resistance, extremely high piezoelectric effect, and high electron mobility, AlN is attractive for the application of high-temperature, high-frequency and high-power devices [9,10]. Moreover, the lattice constant and thermal expansion coefficient of AlN are close to other Ⅲ–Ⅴ materials, thereby making it suitable as a substrate for the epitaxial growth of materials, such as GaN and AlGaN [11]. To fulfill the above application potential, large-size and high-quality AlN single crystals are indispensable. At present, the physical vapor transport (PVT) method, also called the sublimation–recondensation method, has been proven to be the most successful approach for bulk AlN single crystal seed growth [12,13,14,15,16]. In a nitrogen atmosphere, the AlN powder sublimates into gaseous Al and N_2_ at the higher-temperature source zone and the vapor species transport to the lower-temperature crystalline zone, promoting the growth of AlN crystals at the substrate [17,18,19]. However, it is still challenging to grow large-size and high-quality AlN seeds by PVT due to the harsh growth conditions.

A series of studies on the crystal growth of AlN single crystals have been successively reported after the pioneering work of Slack and McNelly [12]. AlN bulk crystals have been grown on the SiC substrate by Nitride Crystals, Ltd. [20,21] and thick AlN bulk crystals with a diameter of 2 inches were grown by the method, but the crystals are rich in C and Si impurities, and the rocking curves exhibit a considerable scatter of FWHMs, up to several hundred arcsec. The growth of the AlN crystals is strongly anisotropic, i.e., a superiority along the c-axis through a spontaneous nucleation process [16,22,23]. Many researchers suggest that the morphology and quality of AlN single crystals is distinct with different growth temperatures and temperature field distributions. The Leibniz Institute for Crystal Growth used spontaneous nucleation and the subsequent freestanding growth method to prepare AlN seed crystals of high structural perfection [22]. Hartmann et al. designed a perforated sheet above the source material inside the TaC crucible, which was used to change the supersaturation of nucleation position. Based on this design, a suitable supersaturation range of 0.25–0.3 [24,25] was obtained through numerical simulation. However, the relatively higher carbon concentration caused by this setup would decrease the quality of grown crystals to some extent [24,25,26]. In numerical simulation, some works have been reported about the effect of the temperature field changes on crystal growth. Cai investigated the effect of graphite insulation degradation during growth on the temperature distribution inside the crucible [27]. Wu investigated the effect of the hot zone structure layout on the temperature distribution in the growth chamber to obtain large-size AlN crystal by FEMAG software [28]. Bei Wu compared the temperature distribution in the growth cell for resistance and induction heating systems by developing a numerical model to simulate two AlN sublimation growth systems. The temperature distribution of the AlN crystal growth chamber is affected by many factors. The simulation of thermal distribution is conductive to increasing the efficiency of experiment [29].

In this work, we report the control of the nucleation rate and the single growth mode of an AlN single crystal on the polycrystalline tungsten substrate, which provides a potential method to prepare large-size and high-quality AlN seed crystals. A detailed two-dimensional model of the crystal growth furnace is built to simulate the thermal distribution of AlN growth considering conduction heat transfer, radiant heat transfer, and heat transfer fluid. On this basis, an optimal axial temperature field distribution and thermal inversion [17] between the sublimation zone and crystallization zone for the best nucleation quality are achieved using a three-zone resistance heating setup. The crucible structure is improved to obtain proper radial thermal distribution which supports the single growth mode.

## 2. Experimental Setup and Numerical Model

**Figure 1:**
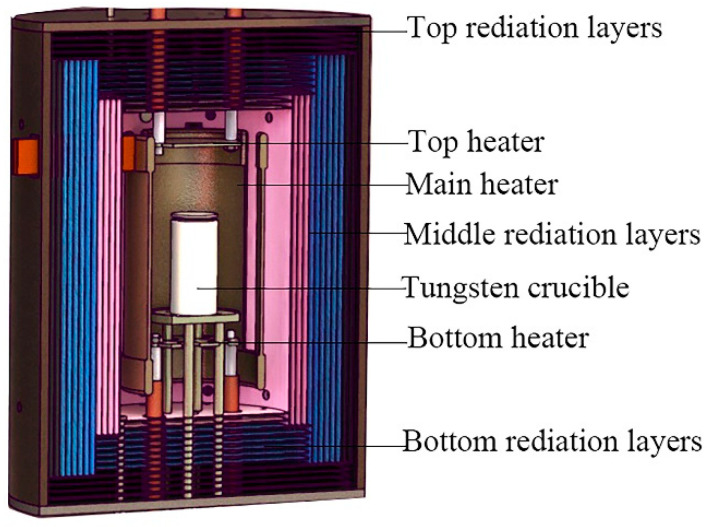
Schematic diagram of AlN crystal-growth furnace.

PVT crystal-growth processes are performed in a tungsten heating element furnace with a resistive heating system. Figure 1 shows a growth unit with three heaters, including top heater used to adjust the thermal field of crystalline zone, main heater used to control the growth chamber temperature, and bottom heater used to compensate the bottom temperature of the material zone. In order to reach a sufficiently high growth temperature of AlN, a multilayer shielded insulation method is used. Three thermocouples for low temperature detection (<1800 °C) and infrared thermometers for high temperature detection (>1000 °C) are installed in three different locations of the growth chamber for temperature detection. The crucible for growing AlN crystal locates at the center of the chamber. The furnace can run under a nitrogen ambience with a pressure of 0.3–5 atm and a high temperature of 2100–2400 °C.

**Figure 2:**
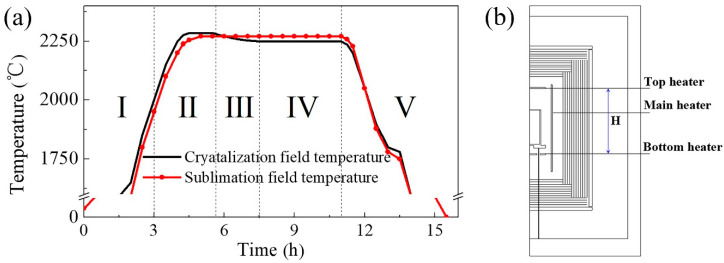
(**a**) Temperature curve of AlN crystals growth; (**b**) Schematic diagram of two dimensional axisymmetric structure of furnace.

All experiments are conducted at 2250–2400 °C under a high-purity nitrogen (99.999%) ambience of 1 atm in the above furnace. The temperature in the growth system can be changed by adjusting the power of heaters. Meanwhile, an appropriate number of radiation shields can prevent heat loss, wasting electronic energy, and consumption of furnace. The axial temperature difference between the highest temperature at the source material (sublimation zone) and the lowest temperature at the substrate (crystallization zone) is the driving force for AlN growth, which can be changed by adjusting the position of the crucible and the power of three heaters. AlN source material at the bottom of the crucible for experiments is prepared by sintering of commercial powder 3–5 times at 1800–2100 °C under a nitrogen ambience of 0.5–1 atm. The growth process of AlN (Figure 2a) can be subdivided into five steps as follow.

(I). Heating by the increasing power of top and main heater
(II). Nucleation suppressing by maintaining negative temperature difference
(III). Nucleation by decreasing the temperature of the crystallization zone
(IV). Crystal growth by maintaining positive temperature difference
(V). Cooling and annealing

An adjustable thermal field is designed to grow high-quality and large-size AlN single crystals. The radial temperature field of the substrate can be regulated by changing the crucible structure. Nevertheless, the absolutely accurate temperature is difficult to obtain because the growth temperature is so high that no detector can measure it directly. Meanwhile, a typical growth cycle lasts for several days, which can cause considerable consumption. Consequently, numerical modeling and simulation have become increasingly essential and indispensable to the understanding and optimization of the crystal growth process.

Numerical simulation has been widely used to study the vapor process because of the difficulty of in situ measurements and the high temperature sensibility of AlN growth. The two-dimensional axisymmetric assumption is used to simplify the practical growth condition in the furnace, as shown in Figure 2b. The main heater has been simulated as a cylindrical tube with the same volume, and the power is substituted for electric current. The crucible height should not exceed the height of growth chamber (H). Holes for the temperature detectors have to be neglected due to the characteristics of the two-dimensional axisymmetric model. Meanwhile, other complex features of the practical furnace have been simplified because of their negligible influence on the thermal distribution. Basically, the model is consistent with the actual furnace. The furnace chamber is filled with nitrogen of an atmospheric pressure. All three forms of heat transfer, including conduction, radiation, and gas convection, are considered. Finite element analysis by COMSOL Multiphysics software is used for thermal field simulation. The initial temperature of the whole device is set to 25 °C. It is worth noting that all the simulation is conducted in a pure nitrogen atmosphere with the pressure of 1 atm when gas convection is taken into account. The physical properties of the materials used for the simulations are presented in Table 1.

## 3. Results and Discussion

### 3.1. Influence of Gas Convection onTemperature Field

**Figure 3:**
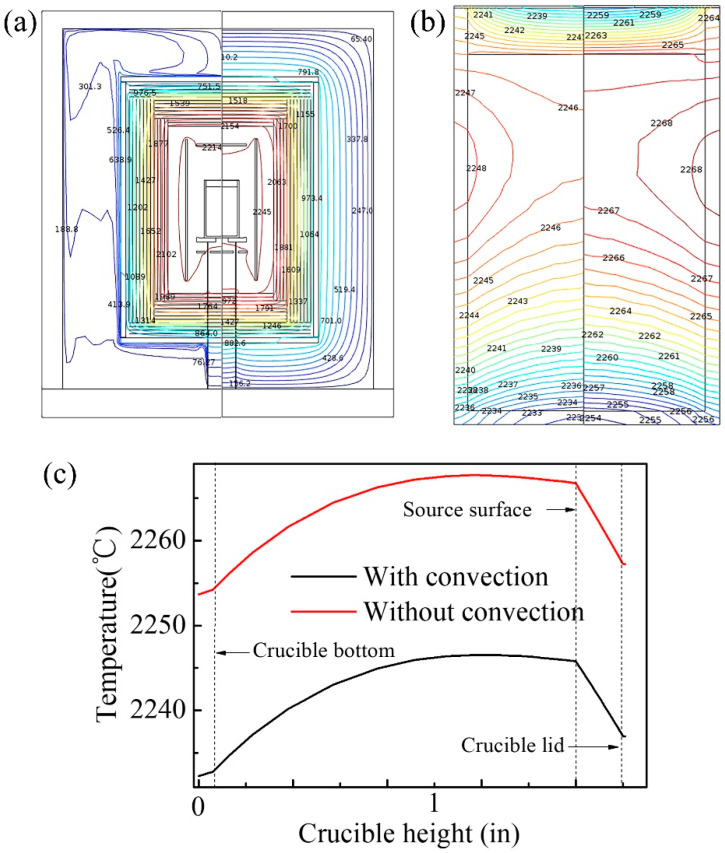
Results with (left) and without (right) gas convection. (**a**) Comparison of the entire equipment temperature distribution; (**b**) Comparison of the temperature isotherms of the tungsten crucible; (**c**) Comparison of the temperature profiles along the axis of symmetry of the crucible.

The effect of gas convection on the temperature field was studied with the power of the top and main heaters both constant. The simulation was performed with or without gas convection, and the results are shown in Figure 3. Figure 3a,b shows the simulated temperature field of the entire equipment and the crucible, respectively. The left halves of the two figures are for the condition with the gas convection, and the right halves for without the gas convection. The two conditions were combined in each figure for an explicit comparison. Figure 3a shows that the temperature under the gas convection condition is lower with a more unordered distribution compared with the no convection. Figure 3b shows the temperature isotherms inside the tungsten crucible, demonstrating a less dependence of the thermal distribution in the crucible on the gas convection. Figure 3c shows the temperature profiles along the axis of symmetry of the crucible with different gas convection conditions. It is clear to see that, under the gas convection condition, the overall temperature in the crucible decreased by 20 °C, with the temperature distribution almost unchanged. After all, the influence of convective heat transfer on the crystal growth can be ignored. So, the convection heat transfer will not be considered in the following to save time and cost of computing.

### 3.2. Influence of Insulating Layer Numbers on Temperature

**Figure 4:**
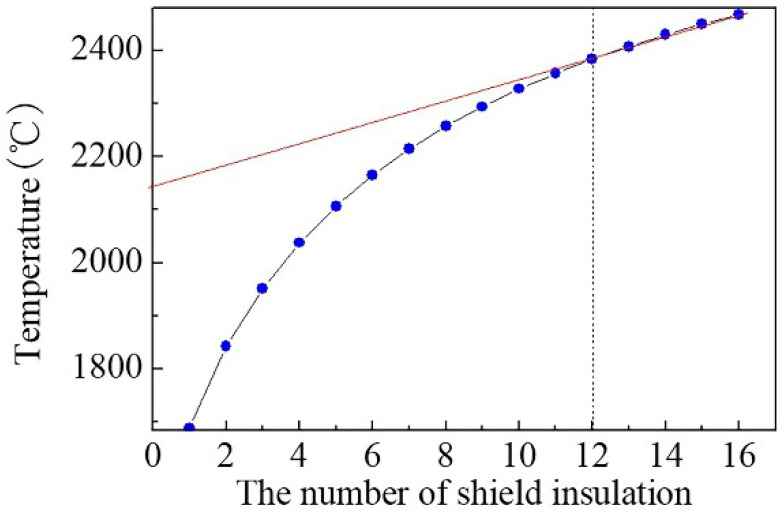
The highest-temperature profile of the crucible with insulation layers with different numbers.

The heating element in the growth furnace generates Joule heat through the resistance heating effect. The heat is transferred to the crucible through heat radiation and convection. The crucible heats the source material through heat conduction. The insulating layers simultaneously reflect and absorb the heat from heating element. The energy absorbed is converted into heat and then radiated again, realizing thermal insulation. Insulating layers of proper material with reasonable quantity can save energy and reduce equipment costs. The rules to select insulating material are low thermal conductivity and high reflectivity (low blackness or emissivity), which are supposed to improve the heat insulation effect. Due to the high temperature of the setup, tungsten is currently selected as the material of the insulation layer. The simulation is performed with different numbers (1~16) of the insulation layer. The results are shown in Figure 4. It can be seen that the temperature change decreases significantly when the number of the insulating layers increases to 12. According to the heat transfer formula [30]

*Q* = A*5.67*e^−8^/(1/*ε_h_* + 1/*ε_c_* − 1)*(*T_h_*^4^ − *T_c_*^4^) (1)

where *A* is surface area of radiation surface, *ε_h_* and *ε_c_* are emissivity of two surfaces, *T_h_* and *T_c_* are temperature of two surfaces, and when the heat transfer with the temperature decreases, the radiation heat transfer becomes worse. Moreover, the temperature would decrease with an increasing interval of the insulation layer. In growth equipment, deformation of the insulation layer caused by the excessive temperature should be considered, so the interval of insulation layers cannot be too small. Moreover, the number of insulation layers should be larger than the theoretical calculated value, to prevent the bond between insulation layers due to the deformation.

### 3.3. The Thermal Distribution Feature of Growth Chamber

**Figure 5:**
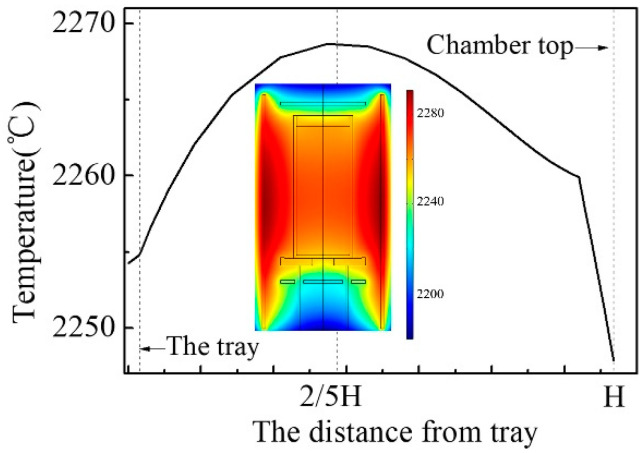
Temperature profile along axis of symmetry of the chamber. Inset displays thermal distribution of the growth chamber.

The main heater controls the overall temperature of the growth chamber, but imposes a limited influence on the temperature distribution in the chamber. The crucible is supposed to cover a suitable area with favorable temperature distribution for crystal growth in the chamber. As the location of crucible is fixed by the location of the tray, a suitable height of the crucible should be grasped through the analysis of thermal distribution features of the growth chamber. Figure 5 shows the thermal distribution in the chamber (the inset) and the temperature profile along with the axis of symmetry of the chamber from the tray. This shows that the lower part of the chamber has a negative temperature difference, which would hinder the crystal growth. The temperature of the chamber bottom can be improved by using a bottom heater. The highest temperature appears at the position of about 2/5H and gradually decreases towards two ends of the chamber. Therefore, the height of the crucible should not be lower than 2/5H. In this paper, we employ the crucible height of 1/2H for following experiment.

### 3.4. Optimization of Axial Temperature Field

**Figure 6:**
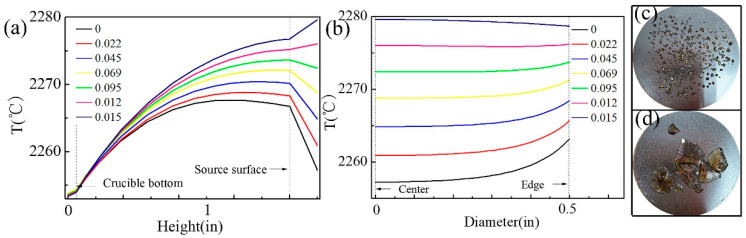
(**a**) Temperature profile along axis of symmetry inside the crucible under different power ratio; (**b**) The radial temperature profile on the substrate under different power ratio; (**c**) The experimental result without nucleation control; (**d**) The experimental result with nucleation control.

The height of the crucible is ascertained according to the temperature distribution of the growth chamber. An appropriate ratio of top-heater power to main-heater power for different stages of crystal growth should be obtained by simulation for the best growing conditions. In order to acquire the longitudinal temperature distribution under different power ratios, the center point of the crucible bottom is used as the reference-temperature point for simulation. The reference temperature is controlled to be a constant value by adjusting the main-heater power under a different top-heater power. Figure 6a shows the axial temperature distribution of the crucible under different power ratios and it shows that the overall temperature slightly rises and the temperature difference (between the source zone and the crystalline zone) decreases as the top-heater power increases. The result shows that the power ratio should be controlled at 0.095 for nucleation stage. And a suitable growth rate can be obtained by slowly reducing the top-heater power value and increasing the main-heater power during the growth stage. The radial temperature distribution is shown in Figure 6b. Figure 6b shows that the temperature distribution on the center region of the substrate is uniform, and the edge of the substrate has a temperature gradient. Experiments with and without inverse temperature field were carried out with the results shown in Figure 6c,d. The sample of Figure 6c,d are defined as A and B respectively. In Figure 6c, AlN crystal nucleation is denser and the size of AlN crystals is smaller in the center region of the substrate than the edge. So, it can be deduced that the nucleation rate and the crystal size are related to the radial gradient. The results agree with reference [31]. In Figure 6a, even if the top-heater power is 0, the temperature of the crucible bottom will still be lower than that of the substrate. During the growth process, the source material will gradually decrease and the generated vapor transports to the top and bottom of the crucible at the same time. As a result, the bottom part of the source material cannot be fully used and the growth rate reduces due to the short supply of Al vapor from the source material to the substrate. Therefore, it is necessary to use a bottom heater to increase the temperature of the crucible bottom. The center point of the crucible top is used as the reference temperature point for simulation, which can be controlled to be a constant value by adjusting the main-heater power under a different bottom heater power.

### 3.5. Optimization of Radial Temperature Field

**Figure 7:**
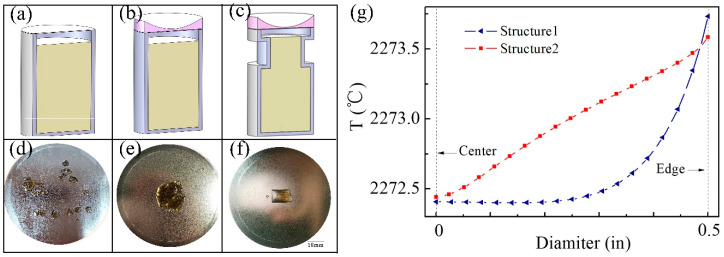
(**a**) The schematic diagram of traditional crucible (structure 1); (**b**) The schematic diagram of crucible with adjusted radial temperature gradient (structure 2); (**c**) The schematic diagram of crucible with adjusted radial temperature gradient and supersaturation (structure 3); (**d**–**f**) The experimental results from three different crucible structure; (**g**) Comparison of radial temperature distribution of the substrate between structure 1 and structure 2.

The density of the AlN crystal nucleus on the substrate is significantly reduced by using the thermal inversion method through different power ratios, but the achievement of a further reduction of nucleation amount and single growth mode also need the adjustment of the radial temperature field. It can be seen from Figure 6b that temperature distribution on the substrate becomes more and more uniform with the increase of the top-heater power. According to the nucleation theory, an atom on the substrate will move after its arrival due to the surface migration energy, and finally settles down in a suitable point for nucleation. The tungsten substrate produced by the powder metallurgic method has a large number of grain boundaries. As shown in Figure 7d, there are a lot of sites on the substrate suitable for nucleation, leading to a random nucleation, which is unfavorable for the growth of single crystals with a large scale. By designing the crucible’s structure and adjusting the radial temperature field of the substrate, the lowest temperature of the substrate will appear in the central region where nucleation preferentially occurs during the nucleation process.

The temperature distribution on the substrate can be changed by using a temperature-adjustment component placed on the top of the traditional crucible. A traditional (structure 1) and a new structure of crucible (structure 2) are shown in Figure 7a,b, respectively. Under the same conditions, the simulations of the temperature distribution of substrate during the nucleation stage were performed in the structure 1 and 2. The results of the radial temperature distributions are shown in Figure 7g. From the center to the edge of substrate, the radial temperature field in structure 1 and 2 show a Box-Cox power exponential and a linear distribution, respectively. The radial temperature gradient of the central area of a substrate is improved in structure 2, which can increase the surface mobility of the atoms on the substrate, and thus increases the radial growth rate to obtain large-size AlN crystals.

In order to ensure a low supersaturation (around 0.1–0.25), the diameter of the upper crucible is decreased to reduce the sublimation of source material during the nucleation stage and increase the temperature gradient during the growth stage. As shown in Figure 7c, the improved structure 3 can achieve better temperature uniformity of the high-temperature region and smaller supersaturation during the nucleation stage than the other two structures.

The crucibles with structure of 1~3 were practically produced and used for experiments at 2300 °C. The results are shown in Figure 7d–f, which are consistent with the results of COMSOL simulation. The nucleation density in Figure 7d is low, but the distribution of the crystals is random. The nucleation in Figure 7e accumulates on the center in spite of several competing nucleus. It can be seen that only one bulk single AlN crystal of about 12 × 7 mm was grown on the center of the substrate using the structure 3, as shown in Figure 7f. The sample of Figure 7f is defined as C. This single growth mode shows a good repeatability, which proves the reliability and feasibility of structure 3. The design of the new crucible structure allows for a low-temperature region on the center of the substrate, enabling the mass transfer of gas species to the center region through the convection and diffusion. Relatively higher supersaturation of Al vapor on the center region benefits for the enlargement of AlN single crystal.

### 3.6. Quality Characterization of AlN Crystal

**Figure 8:**
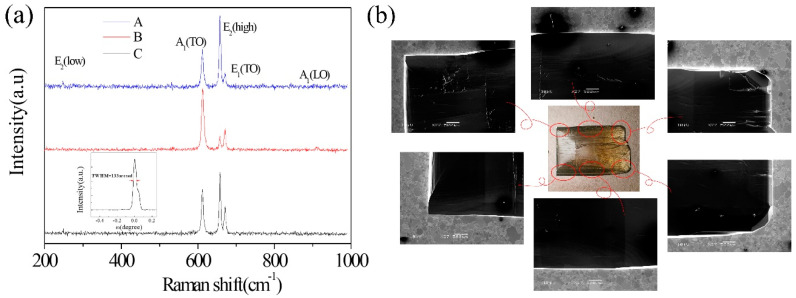
(**a**) Raman spectra of AlN crystals grown with different temperature field. The inset is X-ray rocking curve of sample C; (**b**) Low-magnification SEM image of seed crystal.

To study the effect of the thermal distribution on the quality of AlN crystals, Raman characterization was performed using an AndorSR500 spectrometer with a 532 nm laser as excitation source on AlN crystals (A, B, and C) grown at different temperature field. The Raman spectra are shown in Figure 8a. A_1_(TO), E_2_(high), and E_1_(TO) Raman peaks are obviously observed. The full width at half-maximum (FWHM) of the E_2_(high) mode is generally used to estimate the crystal quality of AlN, and the peak position of E_2_(high) mode is used to determine the stress in the crystal [32]. The peak position of the E_2_(high) mode of the stress-free AlN crystal is 657.4 cm^−1^. For the E_2_(high) mode of three samples (A, B, C), the FWHMs are 5.09, 4.38, and 4.78 cm^−1^ and the peak positions are 657.6, 657.3, and 657.7 cm^−1^, respectively. It can be concluded that the thermal inversion is benefit to the quality of crystal by comparing the FWHM of A and B. The peak position of the E_2_(high) mode of B is closer to the stress-free status. And the quality of C is slightly worse than B as the higher stress in C means more harmful effect on crystal quality. The linear radial distribution for C causes higher stress compared with the Box-Cox power exponential radial distribution for B. The higher stress might lead to more defects such as basal plane dislocations (BPDs) and low angle grain boundaries (LAGBs). It can be concluded that a uniformly distributed temperature field obtained by structure (2) is better to the growth of a stress-free AlN crystal. The inset in Figure 8 is the X-ray diffraction (XRD) rocking curve with Cu Kα radiation on a Philips X-ray diffractometer at 40 kV and 40 mA. A FWHM of around 133 arcsec for the (110) rocking curve is obtained. The FWHMs of rocking curves of AlN bulk crystals grown in Ta crucibles by E.N. Mokhov is over 250 arcsec [20]. The FWHMs of rocking curves of AlN bulk crystals grown on SiC seeds are in the range of 2–5 arcmin [21]. Compared with them, our crystal shows better quality. Figure 8b is a low-magnification SEM image of C sample with a scale bar of 500 μm, revealing the growth direction along an axis.

## 4. Conclusions

The thermal field is studied by theoretical analysis and practical experiments in a PVT AlN crystal-growth furnace. The suitable thermal field and crucible structure are designed to grow high-quality and large-size AlN seed crystals. The nucleation rate is suppressed by using the thermal inversion method which leads to a negative axial temperature gradient in the nucleation suppressing grade and a lower axis and radial temperature gradient in nucleation grade. The low axis temperature gradient can control supersaturation to obtain a low nucleation rate. The relatively low axial and radial temperature gradient benefit for the stress-free crystal growth. Although a uniform temperature zone of the substrate is advantageous to the crystal quality, it is difficult to achieve the enlargement of crystal size. The structure of the crucible is optimized to obtain linear distribution thermal field, which can realize the single growth mode to grow high-quality and large-size AlN single crystals on the polycrystalline tungsten substrate.

## Figures and Tables

**Table 1:** Physical properties of the materials.

	AlN	W	Mu	Al	Stainless Steel
thermal conductivity, k (W m^−1^ k^−1^)	220	175	138	238	44.5
isobaric specific heat, C_p_ (J kg^−1^ K^−1^)	1197	132	250	900	475
Density, ρ (kg m^−3^)	2702	17,800	10,200	2700	7850
Emissivity, ε	0.08	0.04	0.08	0.07	0.85
